# Multicenter Female Fabry Study (MFFS) - clinical survey on current treatment of females with Fabry disease

**DOI:** 10.1186/s13023-016-0473-4

**Published:** 2016-06-29

**Authors:** Malte Lenders, Julia B. Hennermann, Christine Kurschat, Arndt Rolfs, Sima Canaan-Kühl, Claudia Sommer, Nurcan Üçeyler, Christoph Kampmann, Nesrin Karabul, Anne-Katrin Giese, Thomas Duning, Jörg Stypmann, Johannes Krämer, Frank Weidemann, Stefan-Martin Brand, Christoph Wanner, Eva Brand

**Affiliations:** Internal Medicine D, Department of Nephrology, Hypertension and Rheumatology, University Hospital Muenster, Albert-Schweitzer-Campus 1, D-48149 Muenster, Germany; Villa Metabolica, Department for Pediatric and Adolescent Medicine, University Medical Center of the Johannes Gutenberg-University Mainz, Mainz, Germany; Department II of Internal Medicine and center for Molecular Medicine Cologne, University of Cologne, Cologne, Germany; Albrecht-Kossel-Institute for Neuroregeneration, University of Rostock, Rostock, Germany; Department of Medicine, Division of Nephrology, University Hospital Charité, Campus Virchow-Klinikum, Berlin, Germany; Department of Neurology, University of Wuerzburg, Wuerzburg, Germany; Department of Medicine, Divisions of Cardiology and Nephrology, Comprehensive Heart Failure Center, Fabry Center for Interdisciplinary Therapy (FAZIT), University of Wuerzburg, Wuerzburg, Germany; Department of Neuropediatrics and Inborn Metabolic Disorders, Centre for Rare Diseases, University Children’s Hospital, St. Josefs-Hospital, Bochum, Germany; Department of Neurology, University Hospital Muenster, Muenster, Germany; Department of Cardiovascular Medicine, Division of Cardiology, University Hospital Muenster, Muenster, Germany; Department of Pediatrics and Adolescence Medicine, University of Ulm, Ulm, Germany; Katharinen-Hospital Unna, Unna, Germany; Institute of Sports Medicine, Molecular Genetics of Cardiovascular Disease, University Hospital Muenster, Muenster, Germany

**Keywords:** Fabry disease, Females, Lyso-Gb3, Enzyme replacement therapy, Guidelines

## Abstract

**Background:**

The aim of the present study was to assess manifestations of and applied treatment concepts for females with Fabry disease (FD) according to the current European Fabry Guidelines.

**Methods:**

Between 10/2008 and 12/2014, data from the most recent visit of 261 adult female FD patients from six German Fabry centers were retrospectively analyzed. Clinical presentation and laboratory data, including plasma lyso-Gb3 levels were assessed.

**Results:**

Fifty-five percent of females were on enzyme replacement therapy (ERT), according to recent European FD guidelines. Thirty-three percent of females were untreated although criteria for ERT initiation were fulfilled. In general, the presence of left ventricular hypertrophy (LVH) seemed to impact more on ERT initiation than impaired renal function. In ERT-naïve females RAAS blockers were more often prescribed if LVH was present rather than albuminuria. Affected females with missense mutations showed a similar disease burden compared to females with nonsense mutations. Elevated plasma lyso-Gb3 levels in ERT-naïve females seem to be a marker of disease burden, since patients showed comparable incidences of organ manifestations even if they were ~8 years younger than females with normal lyso-Gb3 levels.

**Conclusion:**

The treatment of the majority of females with FD in Germany is in line with the current European FD guidelines. However, a relevant number of females remain untreated despite organ involvement, necessitating a careful reevaluation of these females.

**Electronic supplementary material:**

The online version of this article (doi:10.1186/s13023-016-0473-4) contains supplementary material, which is available to authorized users.

## Background

Fabry disease (FD; OMIM #301500) is an X-linked (Xq22.1) inborn error of glycosphingolipid degradation resulting from deficient α-galactosidase A activity (GLA; 300644) due to mutations in the *GLA* gene. Fabry-specific manifestations result from systemic lysosomal accumulation of mainly globotriaoslyceramide (Gb3) [1]. Gb3 accumulation in cells of different tissues is accompanied by a high risk of early onset of stroke, life-threatening arrhythmia, myocardial infarction, or cardiac and renal failure, leading to a reduced life expectancy [1]. Enzyme replacement therapy (ERT) with recombinant GLA, including agalsidase-alfa (Replagal, Shire) and agalsidase-beta (Fabrazyme, Genzyme) results in subcellular Gb3 clearance, leading to stabilization or at least slowing of disease progression in males and females [2–11]. While the onset of first symptoms (Fabry-associated pain, angiokeratoma, abdominal pain, cornea verticillata, hypo- or anhidrosis) in affected hemizygous males with low or absent enzymatic GLA activity starts in early childhood, heterozygous female patients may display much more variability in disease onset, severity, and progression. Because of this heterogeneous clinical picture in females, the optimal time point for ERT initiation still remains controversial.

Current FD guidelines and recommendations suggest ERT initiation in females with FD after the onset of first FD-typical renal, cardiac, and/or cerebral complications, or in rapidly progressive disease [12, 13]. According to Biegstraaten and colleagues, ERT should be considered in females with classical and non-classical phenotype if albuminuria/proteinuria, an estimated glomerular filtration rate (eGFR) <90 ml/min/1.73 m^2^, cardiac hypertrophy, signs of cardiac rhythm disturbances, cerebral white matter lesions, transient ischemic attack or stroke, FD-related pain or gastrointestinal (GI) symptoms are present [13].

However, since ERT is assumed to be most effective when started early before the onset of fibrosis or other irreversible tissue damage [14–16], this strategy might result in a therapeutic dilemma. Therefore, plasma lyso-Gb3 has been discussed as a prognostic marker for disease severity and progression. While this concept is established in males, the clinical relevance of lyso-Gb3 for female patients is still unclear [17–19].

In the Multicenter Female Fabry Study (MFFS), we retrospectively analyzed a cohort of 261 genetically confirmed adult female FD patients from six German Fabry centers to investigate the current ERT treatment status and the implementation of the latest European FD recommendations [13] concerning treatment strategies.

## Methods

### Study design and patients

Between 10/2008 and 12/2014, 261 genetically confirmed adult female FD patients were consecutively recruited or identified by family screenings (index patients were either affected males or females) at Fabry centers of the University Hospitals in Muenster, Wuerzburg, Mainz, Cologne, Rostock, and the Charité Berlin. Patients were retrospectively analyzed in an open cohort study. A comprehensive diagnostic work-up was performed in all centers including medical history and cardiac, renal, and neurological evaluation. Data documentation followed the clinical practice of the German Fabry Expert Centers for a rare multisystemic disorder. The detailed clinical work-up of patients has been reported elsewhere [20]. Gastrointestinal pain includes abdominal pain, tenesmus, or cramping more than once a week. Diarrhea was defined as ≥1 day/month with three loose bowels or >250 g of stool weight per day. Fatigue was defined by the Fukuda criteria [21]. Cardiac assessment included echocardiography and electrocardiography. Left ventricular hypertrophy (LVH) was defined as a septal diameter of >12 mm. Renal function was quantified by the eGFR using the Chronic Kidney Disease-Epidemiology Collaboration equation (CKD-EPI) [22] and the albumin-to-creatinine ratio (ACR) from spot urine. Renal impairment was defined as eGFR <90 ml/min/1.73 m^2^ according to “Kidney Disease: Improving Global Outcomes” (KDIGO) [23] and European FD [13] guidelines and albuminuria as ACR >30 mg albumin per g creatinine (microalbuminuria, 30–300 mg/g; macroalbuminuria >300 mg/g). All patients underwent neurologic examination and a clinical interview focusing on a history of cerebral stroke or transient ischemic attack (TIA). Additionally, the presence of FD-related pain was investigated [24]. In a subset of patients with a characteristic history of acral pain and/or dysesthesias, small fiber neuropathy (SFN) was diagnosed additionally using quantitative sensory testing (QST) [25] and/or by determination of the intraepidermal nerve fiber density in skin biopsies [26, 27]. Disease severity was assessed using the Mainz Severity Score Index (MSSI) [28]. Additional medication was assessed for the current visit. Renin-angiotensin-aldosterone-system (RAAS) blockers include the prescription of angiotensin-converting enzyme blockers, angiotensin receptor blockers, renin blockers, as well as aldosterone antagonists. Diuretics include the prescription of high ceiling/loop diuretics, thiazides, carbonic anhydrase inhibitors, potassium-sparing diuretics. Analgesic drugs include the prescription of opioids, anticonvulsants, selective serotonin reuptake inhibitors, and non-steroidal anti-inflammatory drugs.

### GLA sequencing, measurement of GLA activity and plasma lyso-Gb3

Genotyping for *GLA* gene mutations was performed by direct sequencing of all seven coding exons including adjacent intron-exons boundaries as previously reported [29].

The following described mutations/variations were detected: p.A20P, p.W24*, p.N34S, p.G35E, p.A37T, p.P40L, p.M42V, p.L45P, p.H46R, p.R49G, p.C53S, p.C63Y, p.I91T, p.C94S, p.R112C, p.R112H, p.R118C, p.A121T, p.S126G, p.S128G, p.L129P, p.L131P, p.A135V, p.N139S, p.A143T, p.G147R, p.Y151*, p.W162G, p.L167Q, p.C172Y, p.Y173*, p.G183S, p.M187R, p.M187V, p.W204*, p.K213M, p.N215S, p.R220*, p.N224S, p.R227Q, p.R227*, p.D231N, p.W236C, p.I242V, p.W262C, p.W262*, p.P265L, p.L268S, p.V269A, p.A288D, p.R301P, p.R301Q, p.R301*, p.D313Y, p.V316G, p.I319T, p.N320I, p.Q321*, p.Q327R, p.Q327E, p.G328R, p.E338X, p.W340*, p.R342Q, p.R342*, p.A350P, p.Q357*, p.W399*, IVS2 + 1G > T, IVS2 + 1G > A, IVS5 + 1G > A, IVS6 + 1G > A. An overview of newly detected *GLA* mutations is presented within the supplement (Additional file 1: Table S1). Females with genetic variants of unknown significance (GVUS) and polymorphisms (i.e. p.R112H; p.N139S; p.A143T; p.D313Y) were excluded (*n* = 37) from all calculations.

Nonsense mutations have been defined as single nucleotide exchanges, resulting in stop codons (termination), deletions or insertions of nucleotides resulting in a frame shift or large deletions within the protein, or splice site mutations, resulting in altered splice products of mRNA. GLA activity in leukocytes was determined using 4-methylumbelliferyl-α-D-galactopyranoside (Santa Cruz Biotechnology, Heidelberg, Germany) as previously described [30]. N­acetylgalactosamine (Santa Cruz Biotechnology) was used as specific inhibitor of endogenous α-galactosidase B activity [31]. GLA enzyme activity was determined as nanomoles (nmol) of substrate hydrolyzed per hour (h) per mg protein and expressed as % of reference. For plasma lyso-Gb3, lyso-ceramide was used as reference (Matreya LLC, Pleasant Gap, PA, USA) and D5-fluticasone propionate (EJY Tech, Inc., Rockville, Maryland, USA) served as internal standard. Plasma lyso-Gb3 levels (reference 0.9–2.3 ng/ml) were measured at the University of Rostock (A. Rolfs), and at the University of Mainz (K. Lackner), Germany. To determine if patients were below or above these reference limits, each individual plasma lyso-Gb3 value was compared with the internal reference at the time-point of lyso-Gb3 determination.

### Statistical analysis

Two-hundred sixty-one patients from the participating FD centers were included in the analysis. If not stated otherwise, continuous variables are expressed as mean with standard deviation or median with range. Categorical data is expressed as numbers and relative frequencies in percent. A quality control of assessed data is given in the supplement (Additional file 2: Table S2). An overall data completeness of 88.9 % was achieved. To deal with missing data, analyses were performed with an as-is state for every parameter. Differences between groups were analyzed with the unpaired Student’s t or Mann-Whitney U test for continuous data, and the Fisher’s exact test for categorical data. Statistical significance was considered at a 2-sided *p* < 0.05. All results are reported with their respective 95 % confidence intervals (CI). SAS version 9.3 (SAS Institute Inc., Cary, North Carolina, USA) and GraphPad PRISM V5.0 software (GraphPad Software Inc., La Jolla, CA, USA) were used for all statistical analyses.

## Results

### Clinical characterization of female patients with Fabry disease

**Fig. 1:**
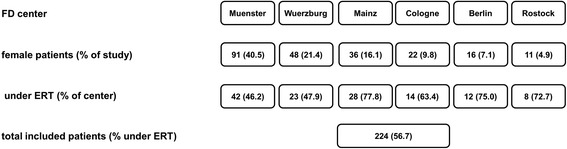
Overview of the participating FD centers and the distribution of female patients. Numbers and frequencies (%) show either distributions within the study (across all centers), or within each participating center

Between 10/2008 and 12/2014 a total of 261 genetically confirmed adult (≥18 years) female FD patients presented at the participating Fabry centers in Muenster, Wuerzburg, Mainz, Cologne, Berlin, and Rostock. Since the presence of GVUS and polymorphisms might influence the outcome, females (*n* = 37) with these variants were excluded (i.e. p.R112H, *n* = 1; p.N139S, *n* = 8; p.A143T, *n* = 18; p.D313Y, *n* = 10). At the time of last visit and data analysis, 127 (56.7 %) females received ERT (i.e. agalsidase-alfa *n* = 108 [85.0 %], or agalsidase-beta *n* = 19 [15.0 %]. The general ERT treatment status differed across centers from 46.2 to 77.8 % females on ERT (Fig. 1).

**Fig. 2:**
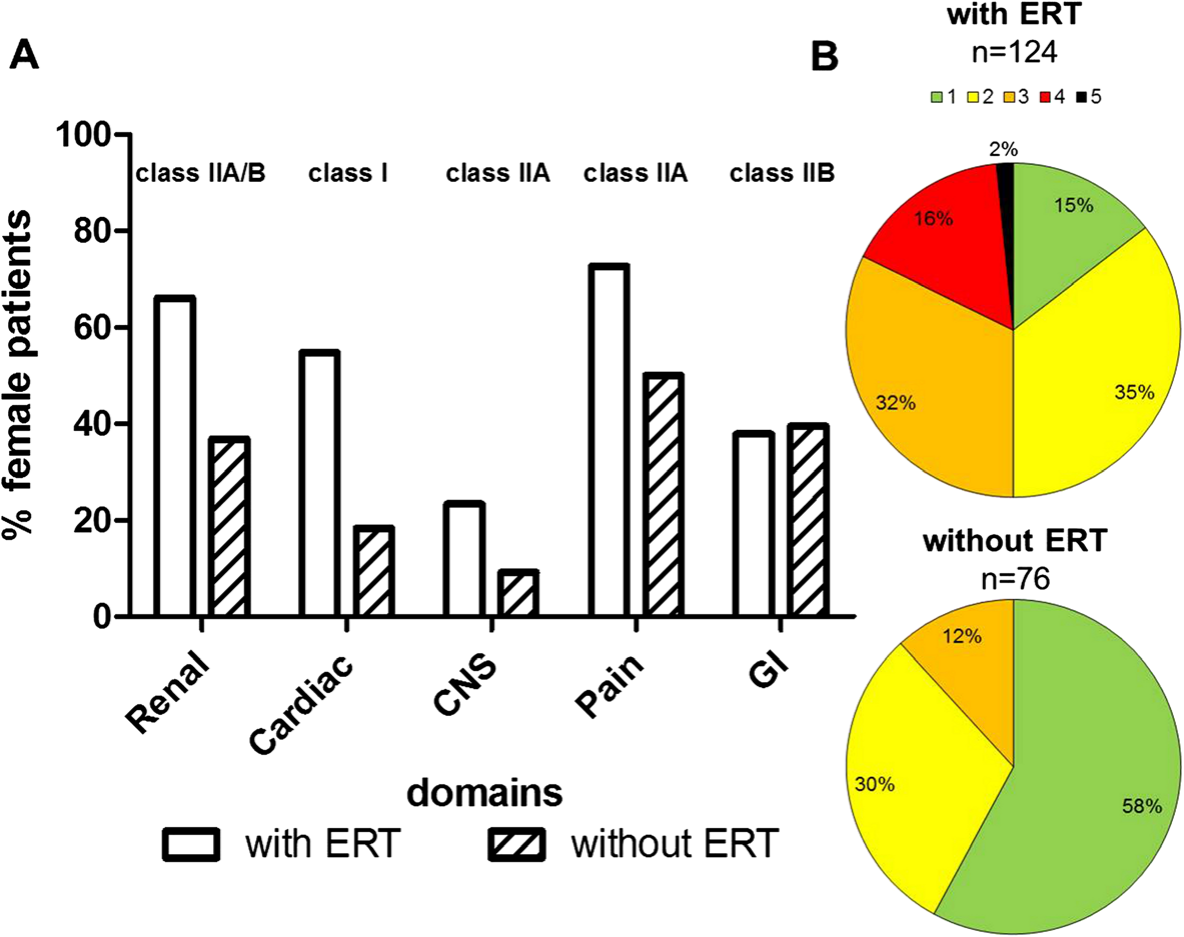
Differences between patients with and without ERT at least one organ manifestation justifying ERT according to current guidelines. **a** Distribution of manifestations in different organs justifying ERT with class I (ERT is recommended) and class IIA/B (ERT should/may be considered) recommendations. **b** Accumulation of different organ manifestations justifying ERT. Females may present with one to five different manifestations (green, yellow, orange, red, black represent 1, 2, 3, 4, 5 simultaneous manifestations, respectively). Cardiac: presence of left ventricular hypertrophy; Renal: eGFR <90 ml/min/1.73 m^2^; CNS: stroke/ transient ischemic attack; Pain: neuropathic/FD-related pain; GI: gastrointestinal symptoms (diarrhea, abdominal pain)

Since the treatment period of patients overlapped with the worldwide agalsidase-beta shortage and a switch of product might have an influence on treatment outcome [11, 20], we assessed all changes in treatment regimens. During agalsidase-beta shortage, nearly all females (18 from 19) under agalsidase-beta received a dose-reduction (0.5 mg/kg body weight) or switched to agalsidase-alfa until re-switch to full agalsidase-beta dose (1.0 mg/kg body weight). From those who received agalsidase-alfa at data assessment (*n* = 108), 20 patients had been switched from agalsidase-beta to -alfa. Consequently, in this “real world” study, both groups were not directly comparable. However, analysis of the clinical presentation revealed that patients under agalsidase-beta tended to be older (*p* = 0.1371) and showed a trend towards a longer ERT duration (*p* = 0.1499) than patients receiving agalsidase-alfa (Additional file 3: Table S3). There were no significant differences between the two patient groups (Additional file 4: Table S4), except for angiokeratoma, NYHA classes, left ventricular diastolic diameter, resulting in a different left ventricular diastolic diameter index (Additional file 4: Table S4). To analyze whether patients were treated in accordance with current European FD guidelines [13] (class I: ERT is recommended; class IIA/B: ERT should/may be considered) at the onset of first organ manifestations, we analyzed the presence of at least one of these manifestations in different organs (cardiac: LVH, renal: eGFR <90 ml/min/1.73 m^2^, CNS: stroke/TIA, pain: FD-related neuropathic pain, gastrointestinal: gastrointestinal symptoms) justifying ERT indication according to classes I to IIA/B [13]. Following this stratification we classified the females into 4 groups: 1) females on ERT with indication (*n* = 124 [55.4 %]), 2) females without ERT with indication (*n* = 76 [33.9 %]), 3) females on ERT without indication (*n* = 3 [1.3 %]), 4) females without ERT without indication (*n* = 21 [9.4 %]) (Table 1, Fig. 2a). Since 34 % of females were not on ERT, although an indication was identified, we further compared treated and untreated females with indications for ERT to identify differences between the two groups. The group of females on ERT (mean treatment duration: 68 ± 48 months) was older, and tended to comprise more patients with nonsense mutations (*p* = 0.0502), more patients with plasma lyso-Gb3 levels above the reference level, and more patients on additional medication (renin-angiotensin-aldosterone-system [RAAS] blockers, diuretics, analgesics) than females not receiving ERT (Table 1). Evaluation of clinical presentation further revealed that female patients on ERT showed a higher frequency of FD-typical signs and symptoms, such as angiokeratoma, edema (ankle), tinnitus, hypacusis, FD-related pain, and fatigue (Table 1). Patients on ERT suffered more often from stroke and demonstrated a higher MSSI score (Table 1). Evaluation of organ manifestations also showed that patients on ERT suffered more often from a cardiac involvement, resulting in higher frequencies of dyspnea and abnormalities in electrocardiogram (ECG) (defined as T-wave negativation). They presented with higher NYHA classes, had increased septal diameters, and higher frequencies for concentric LVH (RR: 1.69 95 % CI 1.37 to 2.08; *p* < 0.0001; Table 2). Renal measures showed that patients on ERT also had a more severe renal impairment in that creatinine- and cystatin C-based eGFR values were severely decreased and showed a difference of >15 ml/min/1.73 m^2^ compared to non-treated female patients (Table 2). Due to the observed differences in eGFR, more patients on ERT were assigned to CKD stage ≥2 than patients not on ERT (RR: 1.70 95 % CI 1.29 to 2.24; *p* = 0.0003; Table 2). Interestingly, the general frequencies of patients with albuminuria were similar, although patients on ERT comprised more patients with macroalbuminuria (Table 2). Females on ERT presented with significantly more manifestations in different organs indicating ERT start [13] than untreated patients (2.5 [1–5] vs 1 [1–3], *p* < 0.0001; Table 2, Fig. 2b).

**Table 1:** Clinical presentation of female Fabry patients

Clinical presentation, laboratory parameters and medication	Females with ERT with indication	Females w/o ERT with indication	Females with ERT without indication	Females w/o ERT without indication	Total
N	124 (55.4)	76 (33.9)	3 (1.3)	21 (9.4)	224
Age [y]	53 ± 14^****^	43 ± 14	44 ± 9	35 ± 13	48 ± 15
Weight [kg]	71.0 ± 17.1	69.9 ± 17.5	66.2 ± 11.2	66.6 ± 19.6	70.1 ± 17.4
Height [cm]	164.5 ± 6.7	165.8 ± 13.8	164.0 ± 4.3	166.7 ± 6.2	165.1 ± 9.1
Heart rate [bpm]	69 ± 13	69 ± 11	63 ± 7	67 ± 8	69 ± 12
SBP [mmHg]	122 ± 17	123 ± 14	126 ± 5	115 ± 16	122 ± 16
DBP [mmHg]	74 ± 9	77 ± 9	76 ± 1	73 ± 10	75 ± 9
Nonsense mutation [n]	59 (50.0)	25 (34.7)	0 (0.0)	4 (19.1)	88 (41.1)
α-galactosidase A activity [% reference]	118 ± 109	118 ± 95	127 ± 74	135 ± 72	119 ± 101
α-galactosidase A activity below reference [n]	42 (56.0)	24 (53.3)	1 (33.3)	3 (42.9)	70 (53.9)
Lyso-Gb3 value [ng/ml]	7.5 ± 6.5	7.9 ± 5.8	4.5 ± 0.3	5.2 ± 4.6	7.4 ± 6.1
Lyso-Gb3 value above reference [n]	70 (94.6)^**^	43 (78.2)	2 (100.0)	9 (69.2)	124 (86.1)
Pts on RAAS blockers [n]	66 (57.4)^****^	16 (23.9)	0 (0.0)	3 (18.8)	85 (42.3)
Pts on diuretic drugs [n]	45 (39.1)^****^	5 (7.7)	0 (0.0)	1 (5.9)	51 (52.5)
Pts on analgesic drugs [n]	37 (35.2)^***^	7 (10.5)	0 (0.0)	0 (0.0)	44 (22.9)
ERT since [months]	68 ± 48	0 ± 0	43 ± 34	0 ± 0	38 ± 49
Angiokeratoma [n]	59 (49.6)^**^	20 (27.4)	2 (66.7)	3 (15.0)	84 (39.1)
Edema [n]	28 (22.6)^****^	1 (1.4)	0 (0.0)	0 (0.0)	29 (13.3)
Gastrointestinal pain [n]	32 (26.2)	26 (36.6)	0 (0.0)	0 (0.0)	58 (27.1)
Diarrhea [n]	33 (27.0)	13 (18.8)	0 (0.0)	0 (0.0)	46 (21.8)
Hypohidrosis [n]	49 (40.5)	22 (29.3)	2 (66.7)	1 (5.0)	74 (33.8)
Cornea verticillata [n]	76 (65.5)	29 (55.8)	2 (66.7)	6 (42.9)	113 (61.1)
Tinnitus [n]	42 (34.2)^*^	13 (18.1)	1 (33.3)	2 (9.5)	58 (26.5)
Hypacusis [n]	27 (22.0)^*^	7 (9.9)	1 (33.3)	0 (0.0)	35 (16.1)
FD-related pain [n]	82 (66.1)^*^	37 (49.3)	0 (0.0)	0 (0.0)	119 (53.6)
Neuropathic pain [n]	40 (32.8)^***^	7 (9.7)	0 (0.0)	0 (0.0)	47 (21.7)
Fatigue [n]	62 (51.2)^****^	15 (20.0)	1 (33.3)	0 (0.0)	78 (35.6)
Ever stroke/TIA [n]	29 (23.4)^*^	7 (9.2)	0 (0.0)	0 (0.0)	36 (16.1)
SFN [n]	22 (29.3)	9 (15.0)	0 (0.0)	2 (12.5)	33 (21.7)
Disease severity score					
MSSI score	20.7 ± 10.8^****^	7.3 ± 5.2	7.3 ± 5.0	1.5 ± 1.5	14.7 ± 11.4

Categorical data are presented as n and are % of total in parenthesis. Otherwise data is presented as mean ± standard deviation. eGFR: estimated glomerular filtration rate (^a^excluding patients with dialysis and/or kidney transplant). DBP: diastolic blood pressure; *MSSI* Mainz Severity Score Index, *Pts* patients, *RAAS* renin-angiotensin-aldosterone-system, *SBP* systolic blood pressure, *SFN* small fiber neuropathy, *TIA* transitory ischemic attack

^*^ *p* < 0.05, ^**^ *p* < 0.01, ^***^ *p* < 0.001, ^****^ *p* < 0.0001

**Table 2:** Cardiac and renal parameters

Cardiac measures	Females with ERT with indication	Females w/o ERT with indication	Females with ERT without indication	Females w/o ERT without indication	Total
Dyspnea [n]	54 (43.6)^***^	14 (18.7)	0 (0.0)	1 (4.8)	69 (30.9)
NYHA class [n]					
none	29 (28.2)	31 (45.6)^*^	1 (50.0)	9 (56.3)	70 (37.0)
I	34 (33.0)	25 (36.8)	1 (50.0)	6 (37.5)	66 (34.9)
II	27 (26.2)	11 (16.2)	0 (0.0)	1 (6.3)	39 (20.6)
III	13 (12.6)^**^	1 (1.5)	0 (0.0)	0 (0.0)	14 (7.4)
IV	0 (0.0)	0 (0.0)	0 (0.0)	0 (0.0)	0 (0.0)
LV diastolic diameter [mm]	45.8 ± 5.5	46.2 ± 4.4	42.5 ± 3.5	46.4 ± 7.2	46.0 ± 5.3
LV systolic diameter [mm]	28.8 ± 6.1	28.5 ± 4.7	24.0 ± 1.4	29.7 ± 4.9	28.7 ± 5.5
LVDDi [cm/m^2^]	2.79 ± 0.33	2.83 ± 0.68	2.58 ± 0.12	2.78 ± 0.42	2.81 ± 0.49
Septal diameter [mm]	12.3 ± 3.6^****^	9.6 ± 2.0	9.5 ± 2.1	8.4 ± 1.5	11.0 ± 3.3
LVH [n]	64 (55.7)^****^	14 (18.9)	0 (0.0)	0 (0.0)	78 (37.0)
Posterior wall diameter [mm]	11.3 ± 3.1^****^	9.4 ± 1.9	10.5 ± 0.7	8.9 ± 1.6	10.4 ± 2.8
RWT [cm]	0.53 ± 0.17^****^	0.41 ± 0.09	0.48 ± 0.11	0.38 ± 0.10	0.47 ± 0.15
ECG abnormalities [n]	42 (36.5)^****^	7 (10.0)	1 (50.0)	1 (5.3)	51 (24.8)
Pacemaker [n]	18 (14.5)^***^	0 (0.0)	0 (0.0)	0 (0.0)	18 (8.0)
Myocardial infarction [n]	7 (6.9)^*^	0 (0.0)	0 (0.0)	0 (0.0)	7 (3.5)
Renal measures					
Albumin/creatinine-ratio [mg/g_Creatinine_]	48 [0–4773]	37 [0–734]	7 [7]	9 [0–226]	39 [0–4773]
Albuminuria [n]	60 (61.9)	30 (58.8)	0 (0.0)	6 (40.0)	96 (58.5)
Microalbuminuria (30–300 mg/g_Creatinine_) [n]	40 (66.7)^*^	27 (90.0)	0 (0.0)	6 (100.0)	73 (76.0)
Macroalbuminuria (>300 mg/g_Creatinine_) [n]	20 (33.3)	3 (10.0)	0 (0.0)	0 (0.0)	23 (24.0)
Cystatin C [mg/l]	1.03 ± 0.36^****^	0.78 ± 0.19	0.65 ± 0.18	0.71 ± 0.09	0.90 ± 0.32
eGFR_cys_ [ml/min/1.73 m^2^]^a^	80.4 ± 26.3^****^	103.6 ± 20.7	116.1 ± 20.3	114.5 ± 13.6	92.6 ± 26.7
Creatinine [mg/dl]	0.99 ± 0.93^***^	0.75 ± 0.15	0.64 ± 0.11	0.65 ± 0.09	0.87 ± 0.71
eGFR_creat_ [ml/min/1.73 m^2^]^a^	81.2 ± 25.3^****^	98.6 ± 19.1	108.8 ± 12.3	113.8 ± 12.6	90.4 ± 24.8
eGFR_creat_ < 90 ml/min/1.73 m^2^ [n]	82 (66.1)^***^	28 (36.8)	0 (0.0)	0 (0.0)	110 (49.1)
Hb [mg/dl]	13.3 ± 1.0	13.4 ± 0.99	13.1 ± 0.31	13.3 ± 1.1	13.3 ± 1.02
Dialysis [n]	2 (1.6)	0 (0.0)	0 (0.0)	0 (0.0)	2 (0.9)
Kidney transplantation [n]	1 (0.8)	0 (0.0)	0 (0.0)	0 (0.0)	1 (0.5)
Manifestations in different organs justifying ERT (per patient) [n]	2.5 [1–5]^****^	1 [1–3]	-	-	2 [0–5]

Categorical data are presented as n and are % of total in parenthesis. Otherwise data is presented as mean ± standard deviation or median [range]. eGFR: estimated glomerular filtration rate (^a^excluding patients with dialysis and/or kidney transplant); *Hb* Hemoglobin. eGFR_creat_ and eGFR_cys_ are calculated via the CKD-EPI formulas according to Levey et al. 2009 and Inker et al. 2012, respectively. LVDDi: left ventricular diastolic diameter index; LVH: left ventricular hypertrophy (>12 mm septal diameter); *NYHA* New York Heart Association, *RWT* relative wall thickness. ^*^ *p* < 0.05, ^**^ *p* < 0.01, ^***^ *p* < 0.001, ^****^ *p* < 0.0001

To further investigate whether patients with nonsense mutations have a higher disease severity, we compared patients with missense and nonsense mutations separately from their ERT status (Table 3). All patients showed at least one manifestation justifying ERT treatment according to current guidelines [13]. Patients on ERT with nonsense mutations had a longer treatment duration than patients with missense mutations (Table 3). Furthermore, patients with nonsense mutations more often had elevated plasma lyso-Gb3 levels and angiokeratoma, and showed a trend towards more edema (ankle) (*p* = 0.0541; Table 3). However, no further significant differences in clinical presentation and cardiac as well as renal measures have been detected (Table 3). Also, patients showed no differences for additional medication with RAAS blockers, diuretics, or analgesics.

**Table 3:** Clinical presentation of females with and without ERT with ERT indications with nonsense and missense mutations

	Females with ERT		Females without ERT	
Clinical presentation, laboratory parameters and medication	Mmissense mutations (*n* = 65)	Nonsense mutations (*n* = 59)	Missense mutations (*n* = 51)	Nonsense mutations (*n* = 25)
Age [y]	53 ± 14	53 ± 15	45 ± 15	40 ± 13
ERT duration [months]	57 ± 47	81 ± 46^**^	-	-
α-galactosidase A activity below reference [n]	24 (60.0)	18 (51.4)	11 (39.3)	13 (76.5)^*^
Lyso-Gb3 value [ng/ml]	6.3 ± 4.1	8.6 ± 8.0	6.9 ± 6.0	9.9 ± 5.0
Lyso-Gb3 value above reference [n]	30 (88.2)	40 (100.0)^*^	26 (70.3)	17 (94.4)
Angiokeratoma [n]	25 (39.7)	34 (60.7)^*^	10 (20.4)	10 (41.7)
Edema [n]	10 (15.4)	18 (30.5)	0 (0.0)	1 (4.2)
Gastrointestinal pain [n]	22 (33.9)	10 (17.5)	19 (40.4)	7 (29.2)
Hypohidrosis [n]	22 (34.4)	27 (47.4)	15 (29.4)	7 (29.7)
Cornea verticillata [n]	37 (63.8)	39 (67.2)	16 (50.0)	13 (65.0)
Tinnitus [n]	23 (35.9)	19 (32.2)	9 (18.8)	4 (16.7)
Hypacusis [n]	19 (24.6)	11 (19.0)	4 (8.3)	3 (13.0)
FD-related pain [n]	41 (63.1)	41 (69.5)	21 (41.2)	16 (66.7)^*^
Fatigue [n]	32 (50.8)	30 (51.7)	11 (21.6)	4 (16.7)
Ever TIA [n]	4 (6.4)	9 (15.8)	2 (4.1)	2 (8.3)
Ever stroke [n]	11 (19.0)	10 (19.6)	3 (5.9)	0 (0.0)
SFN [n]	10 (29.4)	12 (29.3)	3 (7.9)	6 (27.3)
Disease severity score				
MSSI score	21.8 ± 12.0	19.6 ± 9.4	7.5 ± 5.3	6.9 ± 5.1
Cardiac measures				
Septal diameter [mm]	11.8 ± 2.7	12.8 ± 4.4	9.6 ± 2.2	9.6 ± 1.7
LVH [n]	33 (55.0)	31 (56.4)	10 (20.0)	4 (16.7)
Posterior wall diameter [mm]	11.2 ± 2.8	11.5 ± 3.4	9.5 ± 1.9	9.1 ± 1.7
RWT [cm]	0.52 ± 0.14	0.54 ± 0.21	0.42 ± 0.10	0.39 ± 0.07
Pacemaker [n]	10 (15.4)	8 (13.6)	0 (0.0)	0 (0.0)
Myocardial infarction [n]	3 (5.7)	4 (8.3)	0 (0.0)	0 (0.0)
Renal measures				
Albumin/creatinine-ratio [mg/g_Creatinine_]	66 [0–1148]	39 [0–4773]	37 [0–734]	36 [6–535]
Albuminuria [n]	34 (64.2)	26 (59.1)	19 (55.9)	11 (64.7)
Creatinine [mg/dl]	1.07 ± 1.24	0.89 ± 0.34	0.76 ± 0.16	0.74 ± 0.12
eGFR_creat_ [ml/min/1.73 m^2^]^a^	81.3 ± 25.4	81.0 ± 25.3	96.7 ± 19.8	101.0 ± 18.4
Manifestations in different organs justifying ERT (per patient) [n]	2 [1–5]	3 [1–4]	1 [1–3]	1 [1–3]

Nonsense mutations include single nucleotide exchanges, resulting in a stop codon (termination), deletion or insertions of nucleotides resulting in a frame shift or large deletions within the protein, or splice site mutations, resulting in altered splice products of mRNA. Categorical data are presented as n and are % of total in parenthesis. Otherwise data are presented as mean ± SD or median [range]. *eGFR* estimated glomerular filtration rate (^a^excluding patients with dialysis and/or kidney transplant), *LVH* Left ventricular hypertrophy, *MSSI* Mainz Severity Score Index, *SFN* small fiber neuropathy, *RWT* relative wall thickness, *TIA* transitory ischemic attack. eGFR_creat_ is calculated via the CKD-EPI formula according to Levey et al. 2009. missense vs. nonsense mutations: ^*^ *p* < 0.05, ^**^ *p* < 0.01

Untreated patients carrying nonsense mutations were slightly but not significantly younger than patients with missense mutations. Although untreated patients with nonsense mutations demonstrate more often a reduced GLA activity and tended to have higher plasma lyso-Gb3 levels (*p* = 0.0505), no further clinical differences in the group of patients without ERT have been detected (Table 3).

### Plasma lyso-Gb3 in ERT-naïve females with FD

Since the biomarker lyso-Gb3 correlates well with disease manifestation and progression in male FD patients, we analyzed whether differences in plasma lyso-Gb3 levels might also explain differences in FD-specific manifestations and symptoms in female patients. Therefore, we compared ERT-naïve females (independent of a manifestation that would indicate ERT) with lyso-Gb3 levels below the reference value (*n* = 16) with those above the reference value (*n* = 52) (Table 4). Females with lyso-Gb3 levels above the reference were younger than patients with normal lyso-Gb3 levels (41 ± 13 vs. 49 ± 15 years; *p* = 0.0299). Females with elevated lyso-Gb3 levels comprised more patients with nonsense mutations (Table 4). Furthermore, females with elevated lyso-Gb3 levels suffered more often from FD-related pain (Table 4). Interestingly, evaluation of further FD-typical symptoms as well as cardiac and renal measures showed no intergroup differences. Also, the prescription of additional medication was similar in both groups. However, patients with elevated lyso-Gb3 levels tended to a higher albumin-creatinine-ratio (ACR; *p* = 0.0530; Table 4).

**Table 4:** Differences in clinical presentation between ERT-naïve females below and above the plasma lyso-Gb3 reference values

Clinical presentation, laboratory parameters and medication	Females below the reference (*n* = 16)	Females above the reference (*n* = 52)
Age [y]	49 ± 15	41 ± 13^*^
Nonsense mutation [n]	1 (6.3)	19 (36.5)^*^
Lyso-Gb3 value [ng/ml]	0.9 ± 0.5	9.4 ± 5.0^**^
FD-related pain [n]	2 (12.5)	25 (48.1)^*^
Ever TIA/ stroke [n]	3 (18.8)	4 (8.0)
SFN [n]	1 (6.3)	8 (16.7)
Disease severity score		
MSSI Score	4.3 ± 3.8	6.8 ± 5.9
Cardiac measures		
Septal diameter [mm]	9.1 ± 1.4	9.4 ± 2.1
LVH [n]	2 (12.5)	7 (14.0)
Posterior wall diameter [mm]	9.2 ± 1.8	9.4 ± 1.9
RWT [cm]	0.41 ± 0.09	0.40 ± 0.09
Pacemaker [n]	0 (0.0)	0 (0.0)
Renal measures		
Albumin/creatinine-ratio [mg/g_Creatinine_]	9 [5–51]	36 [0–734]
Albuminuria [n]	2 (28.6)	21 (56.8)
Creatinine [mg/dl]	0.76 ± 0.21	0.74 ± 0.13
eGFR_creat_ [ml/min/1.73 m^2^]^a^	96.1 ± 26.2	100.7 ± 18.9
Manifestations in different organs justifying ERT (per patient) [n]	1 [0–3]	1 [0–3]

Categorical data are presented as n and are % of total in parenthesis. Otherwise data is presented as mean ± SD or median [range]. *eGFR* estimated glomerular filtration rate, *LVH* left ventricular hypertrophy, *MSSI* Mainz Severity Score Index, *RWT* relative wall thickness, *SFN* small fiber neuropathy, *TIA* Transitory ischemic attack. ^*^ *p* < 0.05, ^**^ *p* < 0.0001

## Discussion

The aim of our multicenter study was to investigate the current ERT treatment status and the implementation of the European FD guidelines and recommendations in female patients with FD in Germany.

Our main findings are: 1) Fifty-seven % of female FD patients in Germany were under ERT, nearly all (except three females) presented with different organ manifestations that justify ERT initiation according to current European guidelines [13]; 2) one third of females remained without ERT, although indications (organ manifestations) for ERT initiation were fulfilled; 3) the presence of LVH seems to impact more on ERT initiation than impaired renal function; 4) in ERT-naïve females the prescription of RAAS blockers is mostly consistent with the presence of LVH, but not with albuminuria; 5) once affected females with missense mutations show similar disease burden compared to females with nonsense mutations; 6) elevated plasma lyso-Gb3 levels in ERT-naïve females seem to be a marker of disease burden, since these patients showed comparable incidences of organ manifestations and a higher frequency of FD-related pain even if they were ~8 years younger than females with normal lyso-Gb3 levels.

The time point for ERT initiation in males with FD is clinically well established, since patients often suffer from a FD-typical organ involvement and/or display abnormal biomarkers (i.e. low or absent GLA activity, high plasma lyso-Gb3 values). In contrast, the optimal time point for ERT initiation in females with FD, often classified as “only” asymptomatic or minor symptomatic carriers still remains unclear, as their disease manifestations and progression, as well as biomarker levels are diverse.

Most guidelines recommend ERT initiation in females with FD as soon as FD-typical organ manifestations such as LVH, renal insufficiency, or clinical events such as TIA or stroke appear [12, 13]. Our analysis of 224 females with FD may reflect an average of women with FD in Germany. About 55 % of the affected females are on ERT, confirming the prevalence of 53 % reported in the Fabry Outcome Survey (FOS) registry (FOS Investigator report 2012–2013) and suggesting that the reported prevalence of 34 % treated females within the Fabry Registry [32, 33] might be too low, at least for Germany.

Of those patients receiving ERT, nearly all showed at least one organ manifestation defined as LVH, an eGFR <90 ml/min/1.73 m^2^, cerebral complications, pain, or GI symptoms indicating ERT. Concerning this aspect, the treatment concept for females with FD in Germany is in line with the current European FD recommendations and guidelines [13]. Nevertheless, still one third of ERT-naïve female patients suffered from at least one clinically relevant organ manifestation. Even if the observed LVH, renal insufficiency, or stroke and/or TIA might also be due to comorbidities and, more importantly, some patients refuse ERT, or can’t receive ERT due to pregnancy or future family planning, the remaining number of clinically not justifiable untreated affected females seems to be relevant. As mentioned before, one reason for the relatively high frequency of ERT-untreated females with mild renal involvement only might be that many physicians did not recognize these females as patients at risk. In clinical practice, these patients may benefit from RAAS blockers. If under this treatment and with comorbidities/ risk factors (such as arterial hypertension) well-controlled, renal function still declines, ERT should be initiated. The high incidence and increased risk for LVH in patients on ERT indicates that the presence of LVH seems to impact more on ERT initiation than impaired renal function. These observations urge the need for a more stringent implementation of the new European FD guidelines for females across centers and reevaluation of untreated females. Furthermore, some female patients refusing ERT might benefit from future chaperone treatment strategies [34].

Renal biopsies demonstrate that even in children Gb3 accumulation within podocytes affects foot process effacement without presence of albuminuria, indicating an early Fabry nephropathy [35]. In fact, several studies suggest a better benefit when ERT is started at an early disease stage before irreversible organ damage such as fibrosis occurs [9, 14–16]. Interestingly, in females, cardiac replacement fibrosis can already appear at a non-hypertrophic disease stage, being in contrast to males who generally develop LVH prior to replacement fibrosis [36, 37]. In our study, we did not include data on cardiac MRIs, since most of the participating centers routinely perform echocardiography but not cardiac MRI. Of note, in our subset of 68 cardiac MRIs, 41 females revealed late enhancement as a sign of cardiac fibrosis. Interestingly, 22 females with late enhancement did not yet present with LVH. Of these, 12 were not treated with ERT. Hence, in females with FD cardiac MRIs should be routinely performed to identify those with cardiac involvement, necessitating ERT initiation [37].

In addition, protective drugs such as RAAS blockers are needed in females (and males) with renal insufficiency, proteinuria, and LVH. In our study cohort, RAAS blockers were given in >40 % of all females with a >2.0-fold higher treatment frequency in females on ERT compared to those without ERT (57 % vs. 24 %). In general, the frequency of renoprotective and cardioprotective RAAS blocker prescription was comparable across FD centers and in accordance with the frequencies of LVH and albuminuria only in females on ERT. In patients without ERT, a similar relation was found for the presence of LVH and RAAS blockers, but not with albuminuria. Since albuminuria/proteinuria is one of the first signs of impaired renal function, and according to recent KDIGO guidelines [23], persistent albuminuria is associated with poor clinical outcome a more aggressive renoprotective intervention should be considered in these patients.

Interestingly, at the time-point of data assessment, we observed no clinically relevant differences concerning FD-manifestations and disease burden between females receiving either agalsidase-alfa or agalsidase-beta. Although the relative small group of agalsidase-beta-receiving patients might be a limitation, it seems that physicians do not prefer any of these two drugs to treat more severe affected female patients.

Since ERT initiation in female patients based on the clinical presentation might be too late, a prognostic biomarker identifying females with a rapid disease progression is warranted. While plasma lyso-Gb3 seems to be a useful prognostic marker to elucidate disease severity and progression in affected males and correlates well with ERT effectiveness [17, 38, 39], lyso-Gb3 levels are difficult to correlate with disease burden and progression in females and patients with non-classical *GLA* mutations [17–19, 39]. In our study, plasma lyso-Gb3 levels above the reference were more frequent in females on ERT compared to treatment-naïve females. ERT-naïve female patients with elevated lyso-Gb3 levels showed comparable incidences of organ manifestations and a higher frequency of FD-related pain, even if they were 8 years younger than females with normal lyso-Gb3 levels. Therefore, our preliminary data suggest that elevated plasma lyso-Gb3 levels in females also seem to be a marker of disease burden. However, further longitudinal observational studies are warranted to clarify the prognostic value of lyso-Gb3 in ERT-naïve females with different lyso-Gb3 levels.

In contrast to most males, females with FD present with a heterogeneous clinical picture and variable disease progression, independent of the presence of a nonsense or a missense mutation. Our results demonstrate that although females with missense mutations seem to have a lower risk to suffer from severe FD-typical manifestations and events justifying ERT in general, the disease burden in once affected patients is similar, independent of the disease-causing underlying *GLA* mutation (i.e. nonsense or missense). The variation and the missing genotype-phenotype correlation might be due to the controversially discussed X-chromosomal inactivation. While Elstein and colleagues did not find any associations with clinical signs in 77 females FD patients analyzed for skewed X-inactivation [40], Echevarria and colleagues demonstrated significant differences in disease progression depending on the direction and degree of X-inactivation in 56 patients [41]. In that respect, also larger longitudinal studies are warranted to confirm these results and to demonstrate an effect of skewed X-chromosomal inactivation on FD phenotype in affected females.

## Conclusions

We conclude that the treatment concept for females with FD in Germany is in line with the current European Fabry guidelines. However, a relevant number of females remain untreated despite organ involvement, necessitating a careful reevaluation of these females. Elevated plasma lyso-Gb3 levels in females seem to be a marker of disease burden, even if the prognostic value of elevated lyso-Gb3 levels for ERT initiation in females needs evaluation in future observational studies.

## Limitations

The intention of this study was to present a current clinical overview of females with FD and their treatment status and to assess the implementation of current European FD guidelines [13] for ERT treatment. Analysis and readout of techniques such as echoes have been performed within each center by the same on-side specialized operators, which limits intra-center variations. However, slight variations between participating centers cannot be fully excluded. In addition, some parameters such as plasma lyso-Gb3 or GLA activity were not available for the entire study cohort. Since no longitudinal analysis starting with treatment-naïve baselines has been performed and treatment regimens of agalsidase-alfa and -beta groups were heterogeneous, no comparisons of effectiveness between agalsidase-alfa or-beta could be drawn.

## Abbreviations

ACR, albumin/creatinine ratio; CI, confidence interval; CKD, chronic kidney disease; CNS, central nervous system; ECG, electrocardiogram; eGFR, estimated glomerular filtration rate; ERT, enzyme replacement therapy; FD, Fabry disease; Gb3, globotriaosylceramide; GLA, alpha-galactosidase A; GVUS, genetic variance of unknown significance; KDIGO, kidney disease improving global outcomes; LVH, left ventricular hypertrophy; MFFS, Multicenter Female Fabry Study; MSSI, Mainz Severity Score Index; NYHA, New York Heart Association; PCR, polymerase chain reaction; QST, quantitative sensory testing; RAAS, renin-angiotensin-aldosterone-system; RR, relative risk; SFN, small fiber neuropathy; TIA, transient ischemic attack.

## Additional files

Additional file 1: Table S1. Summary of newly identified coding *GLA* mutations and all identified deletions/insertions. (DOC 53 kb) Additional file 2: Table S2. Quality control of assessed data for analyzed patients (*n* = 224). (DOC 33 kb) Additional file 3: Table S3. Differences in clinical presentation between females receiving agalsidase-alfa and -beta. (DOC 40 kb) Additional file 4: Table S4. Differences in cardiac and renal measures between females receiving agalsidase-alfa and -beta. (DOC 38 kb)
