# Supplementary material for: Multicenter Female Fabry Study (MFFS) - clinical survey on current treatment of females with Fabry disease
Source: Orphanet J Rare Dis. 2016 Jun 29;11:88. doi: 10.1186/s13023-016-0473-4 (PMC4928260; doi:10.1186/s13023-016-0473-4)
Supplement: Additional file 1: Table S1. — Summary of newly identified coding GLA mutations and all identified deletions/insertions. (DOC 53 kb) [file 13023_2016_473_MOESM1_ESM.doc]

| **Table S1.** Summary of novel identified coding *GLA* mutations and all identified deletions/insertions. | | | | | | | |  |
| --- | --- | --- | --- | --- | --- | --- | --- | --- |
| **coding mutations** | | | | | | | |  |
| **amino acid** | **cDNA** | **patients [n]** | **mean GLA activity, % WT** | **Lyso-Gb3 value above reference [n]** | | **mean MSSI score** | **treated with ERT [n]** |  |
| p.M76I | c.228G>A | 1 | n.d. | n.d. | | 13 | 1/1 |  |
| p.V137D | c.410T>A | 1 | 147 | 1/1 | | 12 | 1/1 |  |
| p.M187R | c.560T>G | 1 | 407 | 1/1 | | 39 | 1/1 |  |
| p.Y216* | c.648T>A | 2 | n.d. | 2/2 | | 17.5±5.5 | 2/2 |  |
| p.Q280* | c.838C>T | 3 | 28±4 | n.d. | | 2±0 | 0/3 |  |
| p.Q312* | c.934C>T | 2 | 87±22 | n.d. | | 10±5 | 1/2 |  |
| p.I317F | c.949A>T | 1 | n.d. | n.d. | | 31 | 1/1 |  |
| p.L388P | c.1163T>C | 1 | 68 | 1/1 | | 16 | 1/1 |  |
| **deletions/insertions** | | | | | | | |  |
| **position** | |  |  |  |  | |  |  |
| c.35_58del | | 2 | 62±38 | 2/2 | 17±2 | | 2/2 |  |
| c.42_48del | | 1 | n.d. | 1/1 | 26 | | 1/1 |  |
| c.270delC | | 1 | n.d. | 1/1 | 20 | | 1/1 |  |
| c.370_683del | | 2 | n.d. | 2/2 | 30±8 | | 2/2 |  |
| c.521insT | | 2 | n.d. | 2/2 | 13.5±1.5 | | 2/2 |  |
| c.702_709del | | 1 | 212 | 1/1 | 10 | | 0/1 |  |
| c.714ins7bp | | 1 | n.d. | 1/1 | 6 | | 1/1 |  |
| c.718_719delAA | | 2 | 39 | 2/2 | 16.5±11.5 | | 1/2 |  |
| c.756_757delAA | | 3 | 67±20 | 1/1 | 5.3±0.9 | | 0/3 |  |
| c.912delC | | 1 | n.d. | 1/1 | 44 | | 1/1 |  |
| c.1072_1074del | | 1 | 15 | 1/1 | 8 | | 1/1 |  |
| c.1195_1196delTG | | 1 | 266 | 1/1 | 5 | | 0/1 |  |
| c.1208delT | | 1 | n.d. | 0/1 | 4 | | 0/1 |  |
| c.1221delA | | 3 | 57±12 | 2/2 | 16±12 | | 1/3 |  |
| c.1222delA | | 1 | 70 | 1/1 | 18 | | 1/1 |  |
| c.1240_1241delTT | | 1 | n.d. | 1/1 | 10 | | 1/1 |  |
| g.3395_6012del | | 1 | 200 | 1/1 | 16 | | 1/1 |  |
