# Supplementary material for: Multicenter Female Fabry Study (MFFS) - clinical survey on current treatment of females with Fabry disease
Source: Orphanet J Rare Dis. 2016 Jun 29;11:88. doi: 10.1186/s13023-016-0473-4 (PMC4928260; doi:10.1186/s13023-016-0473-4)
Supplement: Additional file 2: Table S2. — Quality control of assessed data for analyzed patients (n = 224). (DOC 33 kb) [file 13023_2016_473_MOESM2_ESM.doc]

| **Table S2:** Quality control of assessed data for analyzed patients (n=224). | | |
| --- | --- | --- |
| **Organ/domain** | **Investigation/ measure, n (%)** | **Combined work-Up (%)** |
| **Medical history** | age: 224 (100.0); weight: 224 (100.0); height : 224 (100.0); heart rate: 214 (95.5); SBP: 211 (94.2); DBP: 213 (94.2); ERT status: 224 (100.0) | 97.7 |
| **Laboratory parameters** | genotype: 224 (100.0); GLA activity: 128 (57.1); plasma lyso-Gb3: 144 (64.3) | 73.8 |
| **Additional medication** | RAAS blockers 201 (89.7); diuretic drugs: 200 (89.3); analgesic drugs: 192 (85.7) | 88.2 |
| **Clinical presentation** | angiokeratoma: 215 (96.0); edema: 218 (97.3); gastrointestinal pain: 214 (95.5); diarrhea: 211 (94.2); hypohidrosis: 219 (97.8); cornea verticillata: 185 (82.6); tinnitus: 219 (97.8; hypacusis: 217 (96.9); FD-related pain: 222 (99.1); neuropathic pain: 217 (96.5); fatigue: 219 (97.8); ever stroke: 214(95.5); ever TIA: 216 (96.4); SFN: 152 (67.9); MSSI scores: 209 (93.3) | 93.6 |
| **Cardiac measures** | dyspnea: 223 (99.6); NYHA class: 189 (84.4); LV diastolic diameter: 199 (88.8); LV systolic diameter: 174 (77.7); LVDDi: 197 (87.9); septal diameter: 211 (94.2); LVH: 211 (94.2); posterior wall diameter: 190 (84.8); RWT: 198 (88.4); ECG: 206 (92.0); pacemaker: 224 (100.0); myocardial infarction: 197 (87.9) | 90.0 |
| **Renal measures** | ACR: 164 (73.2); cystatin C: 167 (74.6); eGFR_cys_: 167 (74.6); creatinine: 222 (99.1); eGFR_creat_: 222 (99.1); hemoglobin: 222 (99.1); dialysis: 223 (99.6); kidney transplantation: 224 (100.0) | 89.9 |
| **Overall data completeness** |  | **88.9** |
| ACR: albumin/creatinine-ratio; DBP: diastolic blood pressure; eGFR: estimated glomerular filtration rate; LVDDi: left ventricular diastolic diameter index; LVH: left ventricular hypertrophy; MSSI: Mainz Severity Score Index; NYHA: New York Heart Association; RAAS: renin-angiotensin-aldosterone-system; RWT: relative wall thickness; SBP: systolic blood pressure; SFN: small fiber neuropathy; TIA: transitory ischemic attack | | |
