# Supplementary material for: Multicenter Female Fabry Study (MFFS) - clinical survey on current treatment of females with Fabry disease
Source: Orphanet J Rare Dis. 2016 Jun 29;11:88. doi: 10.1186/s13023-016-0473-4 (PMC4928260; doi:10.1186/s13023-016-0473-4)
Supplement: Additional file 3: Table S3. — Differences in clinical presentation between females receiving agalsidase-alfa and -beta. (DOC 40 kb) [file 13023_2016_473_MOESM3_ESM.doc]

| **Table S3**. Differences in clinical presentation between females receiving agalsidase-alfa and -beta. | | |
| --- | --- | --- |
| **Clinical presentation, laboratory parameters and medication** | **Agalsidase-alfa**  **(n=108)** | **Agalsidase-beta**  **(n=19)** |
| Age [y] | 52±14 | 57±12 |
| Nonsense mutation [n] | 49 (47.6) | 10 (55.6) |
| α-galactosidase A activity [% reference] | 121±116 | 104±63 |
| α-galactosidase A activity below reference [n] | 36 (57.1) | 7 (46.7) |
| Lyso-Gb3 value [ng/ml] | 7.4±7.0 | 7.6±3.0 |
| Lyso-Gb3 value above reference [n] | 60 (93.8) | 12 (100.0) |
| Pts on RAAS blockers [n] | 53 (53.0) | 13 (72.2) |
| Pts on diuretic drugs [n] | 36 (36.0) | 9 (50.0) |
| Pts on analgesic drugs [n] | 31 (33.7) | 6 (37.5) |
| ERT since [months] | 65±50 | 82±35 |
| Angiokeratoma [n] | 57 (53.8) | 4 (25.0)^a^ |
| Edema [n] | 24 (22.2) | 4 (21.1) |
| Gastrointestinal pain [n] | 27 (25.0) | 5 (29.4) |
| Diarrhea [n] | 28 (26.7) | 6 (31.6) |
| Hypohidrosis [n] | 41 (38.7) | 10 (55.6) |
| Cornea verticillata [n] | 67 (67.0) | 11 (57.6) |
| Tinnitus [n] | 35 (32.7) | 8 (42.1) |
| Hypacusis [n] | 22 (20.6) | 6 (31.6) |
| FD-related pain [n] | 67 (62.0) | 15 (79.0) |
| Fatigue [n] | 57 (53.3) | 6 (35.3) |
| Ever TIA [n] | 9 (8.7) | 4 (21.1) |
| Ever stroke [n] | 19 (18.6) | 3 (20.0) |
| SFN [n] | 18 (28.1) | 4 (33.3) |
| **Disease severity score** |  |  |
| MSSI Score | 20.2±11.3 | 21.4±8.6 |
| Categorical data are presented as n and are % of total in parenthesis. Otherwise data are presented as mean ± standard deviation. DS3: Disease severity scoring system; MSSI: Mainz Severity Score Index; Pts: patients; RAAS: Renin-Angiotensin-Aldosterone-System; SBP: Systolic Blood Pressure; SFN: small fiber neuropathy; TIA: transitory ischemic attack.  ^a^p<0.05 | | |
