# Supplementary material for: Multicenter Female Fabry Study (MFFS) - clinical survey on current treatment of females with Fabry disease
Source: Orphanet J Rare Dis. 2016 Jun 29;11:88. doi: 10.1186/s13023-016-0473-4 (PMC4928260; doi:10.1186/s13023-016-0473-4)
Supplement: Additional file 4: Table S4. — Differences in cardiac and renal measures between females receiving agalsidase-alfa and -beta. (DOC 38 kb) [file 13023_2016_473_MOESM4_ESM.doc]

| **Table S4**. Differences in cardiac and renal measures between females receiving agalsidase-alfa and -beta. | | |
| --- | --- | --- |
| **cardiac measures** | **Agalsidase-**  **alfa**  **(n=108)** | **Agalsidase-beta**  **(n=19)** |
| NYHA class [n]  0  I  II  III  IV | 30 (33.7)  31 (34.8)  18 (20.2)  10 (11.2)  0 (0.0) | 0 (0.0)^a^  4 (25.0)  9 (56.3)  3 (18.8)  0 (0.0) |
| LV diastolic diameter [mm] | 45.4±9.5 | 48.4±4.8^a^ |
| LV systolic diameter [mm] | 28.4±5.5 | 30.2±8.9 |
| LVDDi [cm/m²] | 2.76±0.32 | 2.98±0.32^a^ |
| Septal diameter [mm] | 12.3±3.7 | 12.3±3.2 |
| LVH [n] | 56 (55.5) | 8 (50.0) |
| Posterior wall diameter [mm] | 11.3±3.0 | 11.6±3.6 |
| RWT [cm] | 0.53±0.18 | 0.50±0.12 |
| ECG abnormalities [n] | 35 (35.0) | 8 (47.1) |
| Pacemaker [n] | 16 (14.8) | 2 (10.5) |
| Myocardial infarction [n] | 6 (6.8) | 1 (6.7) |
| **renal measures** |  |  |
| Albumin/creatinine-ratio [mg/g_Creatinine_] | 41 [0-2788] | 111 [2-4773] |
| Albuminuria [n]  Microalbuminuria (30-300 mg/g_Creatinine_) [n]  Macroalbuminuria (>300 mg/g_Creatinine_) [n] | 50 (58.1)  34 (68.0)  16 (32.0) | 10 (83.2)  6 (60.0)  4 (40.0) |
| Cystatin C [mg/l] | 0.99±0.34 | 1.17±0.43 |
| eGFR_cys_ [ml/min/1.73 m²]* | 83.0±26.7 | 71.4±24.9 |
| Creatinine [mg/dl] | 0.98±0.98 | 0.95±0.46 |
| eGFR_creat_ [ml/min/1.73 m²]* | 82.1±25.6 | 79.9±24.8 |
| Dialysis [n] | 1 (0.9) | 1 (5.3) |
| Kidney transplantation [n] | 0 (0.0) | 1 (5.3) |
| Categorical data are presented as n and are % of total in parenthesis. Otherwise data are presented as mean ± standard deviation. LVH: Left ventricular hypertrophy (>12 mm septal diameter); LVDDi: Left ventricular diastolic diameter index. NYHA: New York Heart Association; RWT: relative wall thickness. eGFR: estimated glomerular filtration rate (*excluding patients with dialysis and/or kidney transplant); Hb: Hemoglobin. eGFR_creat_ and eGFR_cys_ are calculated via the CKD-EPI formulas according to Levey et al. 2009 and Inker et al. 2012, respectively. ^a^p<0.05 | | |
